# Parametric Dense Stereovision Implementation on a System-on Chip (SoC)

**DOI:** 10.3390/s120201863

**Published:** 2012-02-10

**Authors:** Alfredo Gardel, Pablo Montejo, Jorge García, Ignacio Bravo, José L. Lázaro

**Affiliations:** 1 Electronics Department, University of Alcala, Alcalá de Henares, Madrid 28871, Spain; E-Mails: jorge.garcia@depeca.uah.es (J.G.); ibravo@depeca.uah.es (I.B.); lazaro@depeca.uah.es (J.L.L.); 2 Higher Polytechnic Institute José Antonio Echeverría (CUJAE), La Habana, 19390, Cuba; E-Mail: pablo.montejo@electrica.cujae.edu.cu

**Keywords:** stereovision, reconfigurable hardware, correspondence, entropy, correlation, real-time processing

## Abstract

This paper proposes a novel hardware implementation of a dense recovery of stereovision 3D measurements. Traditionally 3D stereo systems have imposed the maximum number of stereo correspondences, introducing a large restriction on artificial vision algorithms. The proposed system-on-chip (SoC) provides great performance and efficiency, with a scalable architecture available for many different situations, addressing real time processing of stereo image flow. Using double buffering techniques properly combined with pipelined processing, the use of reconfigurable hardware achieves a parametrisable SoC which gives the designer the opportunity to decide its right dimension and features. The proposed architecture does not need any external memory because the processing is done as image flow arrives. Our SoC provides 3D data directly without the storage of whole stereo images. Our goal is to obtain high processing speed while maintaining the accuracy of 3D data using minimum resources. Configurable parameters may be controlled by later/parallel stages of the vision algorithm executed on an embedded processor. Considering hardware FPGA clock of 100 MHz, image flows up to 50 frames per second (*fps*) of dense stereo maps of more than 30,000 depth points could be obtained considering 2 Mpix images, with a minimum initial latency. The implementation of computer vision algorithms on reconfigurable hardware, explicitly low level processing, opens up the prospect of its use in autonomous systems, and they can act as a coprocessor to reconstruct 3D images with high density information in real time.

## 1. Introduction

In recent years, stereovision has become a very attractive sensing technique for obtaining 3D information [1–3]. Recovering depth via triangulation is present in many computer vision systems. Several authors have attempted to imitate the human vision in different electronic systems devoted to stereo vision [2]. Stereovision systems can provide accurate real-time data in different applications. A rough division may be done to differentiate the recovery of 3D measurements using triangulation from different points of view.

The arrangement of the cameras imposes some restrictions on any stereovision algorithm. Typical setups include two or three cameras (ideally coplanar) not too far apart to facilitate the overlap of their images to provide an accurate 3D measurement. Other systems try to obtain a broader 3D measurement from a large covered area, so the cameras are placed throughout a room with a large field of view (for example, for object location in sports video sequences). In our case, the SoC implementation is more suitable to coplanar (compact) stereovision systems. In other stereovision systems in which optical axes intersect, a previous rectification process is needed.

**Figure 1.**
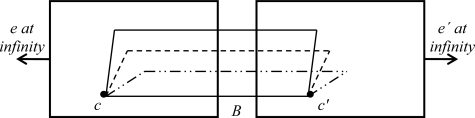
Epipolar geometry.

This type of stereovision systems are ruled by the epipolar geometry which is the intrinsic projective geometry existing between two views from the camera arrangement. A widely used configuration of that geometry is shown in Figure 1 [4], where the cameras with centers (*c*, *c*′) share the same image plane (parallel optics axes), with the epipoles (*e*, *e*′) located at infinity [5,6]. Parameter *B* denotes the distance between optical centers.

In order to obtain 3D measurements is necessary to know the intrinsic parameters of each camera and the extrinsic parameters of the overall stereo system. Typically, these parameters are obtained through a calibration process. The calculation of a 3D measurement involves obtaining the inverse process of image formation expressed in Equation (1):

(1) $$\left[\begin{array}{c} s\left(u_{o}-u\right)\\ s\left(v_{o}-v\right)\\ s \end{array}\right]=\left[\begin{array}{llll} f & 0 & 0 & 0\\ 0 & f & 0 & 0\\ 0 & 0 & 1 & 0 \end{array}\right]\;\left[\begin{array}{cccc} R_{11} & R_{12} & R_{13} & T_{x}\\ R_{21} & R_{22} & R_{23} & T_{y}\\ R_{31} & R_{32} & R_{33} & T_{z}\\ 0 & 0 & 0 & 1 \end{array}\right]\;\left[\begin{array}{c} X_{w}\\ Y_{w}\\ Z_{w}\\ 1 \end{array}\right]$$

where:

- [X_w_Y_w_Z_w_]′ are the 3D coordinates for a considered scene point.
- R is the rotation matrix and T the translation vector.
- *f* is the equivalent focal length of the system.
- s is an scale factor, considering that s = Z_c_.
- (u_o_, v_o_) is the optical center (expressed in pixels).
- (u, v) are pixel coordinates of the point captured by the camera sensor.

**Figure 2.**
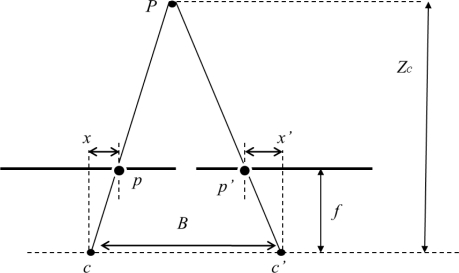
Disparity measurement.

So given a point in an image, possible location correspondence in the other image is limited to a single line. Ideally for two identical aligned cameras it is the same horizontal line. In addition, inside that line only a range of pixels correspond to the overlap between the two cameras field of view. Therefore, to get the depth, $Z_{C}=\frac{Bf}{d_{x}}=\frac{Bf}{x-x^{\prime }}$, it is only necessary to obtain the disparity *d_x_* between corresponding image points, the other parameters remain constant, as shown in Figure 2.

A stereovision system has to solve two problems point selection and correspondences, as not all points of the scene are suitable to find its correspondence. Reliable 3D points are scene points which have features that uniquely identify it in the different images. The other points should be discarded as they would produce mismatches.

Obtained the 3D point correspondences, a whole reconstruction of the 3D data should be done, imposing different rules to give robustness to the image depth of the scene, as described in [7]. The latter is beyond the scope of our paper, restricting the SoC processing to obtain 3D data to later stages of an algorithm.

Many point selection and correspondence methods are used in the scientific literature. A ranking of different stereoscopic algorithms is given in [1]. For the selection of image points, many articles suggest methods for detection of lines and corners in stereo images [7]. This method provides reliable 3D data by finding correspondences for a small area in the stereo images considering the image gradient as a feature of matching. To consider other textured objects, several authors have introduced, in the point selection, the entropy feature of the neighborhood area. Thus, pixels characterized by a high entropy value are more likely to be effective to achieve the correspondence between the images as it is shown in [8]. In our case, the latter method is preferred for point selection so dense 3D data can be recovered, without further exploration about subpixellic location.

For this reduced number of points, a correspondence between images should be obtained thus providing a disparity and also a depth measurement. There are different algorithms and techniques for matching corresponding areas as shown in [9]. Considering a coplanar stereovision system, there is small change of perspective between cameras, so it could be assumed that the object (if not occluded) is captured very similarly by the camera set. To calculate the similarity of two image areas, the most commonly used method is the sum of squared differences (SSD), which in the ideal case of complete correspondence between two areas, SSD is zero. In this case, the algorithm considers there is a correspondence if a minimum SSD exists. Coplanar geometry facilitates the correspondence because matching areas are searched on a line. Computation of SSD is done on selected points around that line. To consider a zone as a correspondence area, that SSD value must be the minimum value of SSD on the line and always below a maximum value threshold. If there are other possible matching areas on the line, an ambiguity arises, and different approaches may be taken into account. In our case, areas with ambiguity in the correspondence are discarded.

Other authors prefer to use the sum of absolute differences (SAD) [1–3,5–8]. The SAD method is hardware efficient, given its regular structure and large parallelism [10]. In contrast, this method introduces some errors if there is any occlusion or light is not uniform [11]. There are other studies that seek to increase the accuracy and reliability of stereovision, using hierarchical Gaussian basis functions and wavelet transform to obtain correspondences [7].

The approach followed in this work is to obtain an autonomous stereovision system, which provides a 3D image of the scene, without limiting the number of correspondences, a common issue of many embedded algorithms that use stereovision. Besides, vision sensors have an increasing number of pixels, thus a SoC for stereovision is more needed than ever. The use of large images is important because the reliability of the depth estimation is highly dependent on the resolution of the input images [2].

Most real time vision systems are expensive, inflexible, with few possibilities for reuse in the design and parameterization. The use of FPGAs can improve these aspects as it is shown in [11] by leveraging the parallelism inherent in many vision algorithms. Many authors have used reconfigurable hardware to accelerate certain computer vision algorithms as in [12].

The paper is organized as follows: Section 2 presents different related works. Section 3 describes the overall architecture; In Section 4, the entropy measurement and entropy block are presented. Section 5 explain data arbitration used in the proposed architecture. In Section 6, it is presented the correlation block with special interest in the efficient control implemented. Finally, results and possible configurable implementations are given in Section 7.

## 2. Related Work

Different authors have proposed architectures to accelerate the computation of dense disparity maps in real-time applications, trying to maintain the accuracy of the results. In [13], a stereovision system using semi-global matching (*SGM*) implement different steps for 3D recovery, from image rectification, to obtaining an estimated disparity map. It achieves a processing framerate of 30 *fps* for VGA images. It is stated there in that local stereo matching methods are more suitable for hardware implementations.

The most common implementations that can be found in the field of dense disparity maps architectures are based on SAD [14–18]. In [16–18] only the SAD process is implemented. In [16] three different configurations are presented, based on the number of resources used. In [14] a stereovision system using three cameras resolves more reliably ambiguous points, making the system more robust, since for each 3D scene point two different correspondences are obtained. However the setup and cost of this stereovision system is greater. In [15], an architecture for stereo matching is based on adaptive ROI in SAD process. The requirement of storage of a sub-image in memory does not allow on-the-fly processing.

Other authors [19,20] propose the use of census transform to calculate the disparity image. The census transform reduces size memory and accesses to that memory. Thus, only binary operations are performed, reducing the processing time, but accuracy is also reduced.

We propose a novel architecture that selects the most appropriate points by an entropy measurement. The configuration allows for different number of candidates, thus there will be a large number of distributed points throughout the image. Image processing is performed on-the-fly, so data storage is reduced at minimum. The main goal is to increase processing speed using minimum hardware; the improvement in accuracy of the stereovision algorithm is out of the scope of our paper, without performing any 3D scene reconstruction, thus high level algorithms will be responsible for improving and interpreting the 3D data results similar to the approach given in [21].

## 3. SoC Architecture for 3D Dense Stereovision

The algorithm proposed herein is divided into the following main blocks:

- Entropy block to select suitable points: This block indicates if a point is suitable for use in a matching process. Corresponding region of interest (ROI) is written in a FIFO memory. In this step, the entropy function is used, as it provides general information without loss of relevant information to obtain scene 3D points.
- ROI correlation matching: In this block it is evaluated a number of areas to look for correspondence, using SSD or SAD. Subsequently, the depth calculation is performed, discarding possible ambiguities.

There is one important pre-requisite for the architecture of the system that is not to store the whole image in memory, to process the image flow on-the-fly and at the full rate of the camera. The producer-consumer architecture should overlap the operation with minimal resources using a double buffer strategy, as will be described later. The architecture uses the same kernel size for the calculation of entropy and correlation matching, thus disparity is calculated for the ROI center.

**Figure 3.**
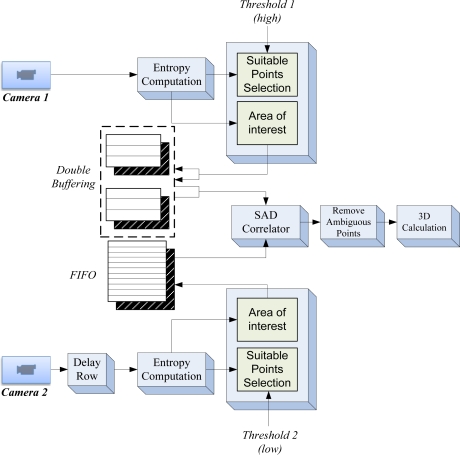
Proposed SoC architecture.

The flowchart shown in Figure 3 presents the overall SoC architecture designed. The procedure starts with the computation of image entropy from both video-camera flows. Using these entropy values, suitable candidate areas of each image are selected, in order to do a ROI matching by the correlator block. From the *N* possible set of cameras of the stereoscopic system, the herein described algorithm process two image flows. A higher threshold for the entropy values is applied to camera 1, selecting ROIs with high entropy values. The threshold is dynamically adjusted in order to maintain a certain percentage of candidate points in the video flow. The neighborhood of candidate points (ROI values) is stored in a local memory to later processing. On the other side, the entropy threshold for the camera 2 image flow, is lower than threshold applied to camera 1, thus correspondence areas are maintained in the processing to match good candidate points selected on camera 1, without losing important image information.

A sub-sampling in possible candidate points is done. Thus if a point is selected as candidate, the next candidate should be at a minimum distance from it, giving a dispersion of candidate points throughout the image. This spreading reduces the amount of local memory needed, allowing the hardware to process the image flow on-the-fly. The high-entropy ROIs from camera 1 image flow are stored in a double buffer memory bank, used to process different image rows. It is worth to note that spreading candidate points in vertical could lead to process non-consecutive rows. The double buffer allows for the candidate selection in one row while making the correlation matching with areas from camera 2 for a previous row. Thus, the image flow from camera 2 should be delayed one complete image line, in order to start providing candidate correlation matching areas to be processed with candidate point areas from camera 1 for a particular line. In the next sections, the hardware implementation of the different blocks is described.

## 4. Entropy Computation

Several authors have identified the entropy value of a ROI area as a suitable indicator to carry out a reliable search of its correspondence in stereo images [8]. The entropy of a ROI is an energy measurement which should be present for a robust matching. The entropy E of a *n*x*m* ROI is calculated using the Equation (2):

(2) $$E\left(i,j\right)=-\sum_{r=0}^{r=n-1}\sum_{q=0}^{q=m-1}P\left(i-r,j-q\right)\;{\text{log}}_{2}\;P\left(i-r,j-q\right)$$

where *P* is the probability that a given gray level appears in the area of size *n* x *m* pixels referenced to the pixel under analysis (coordinate *i,j*). The probability *P* should be obtained as the number of occurrences of each gray level in the area under analysis normalized by the total number of pixels, thus a common formula is based on histogram values as expressed in Equation (3):

(3) $$E=-\sum_{g=0}^{g=\mathrm{Bins}-1}H(g)\;\log_{2}(H(g))$$

where *H(g)* is the histogram binned in a total number of *Bins* and *g* is the current bin gray level in the area of size *n* x *m* pixels referenced to the pixel under analysis.

**Figure 4.**
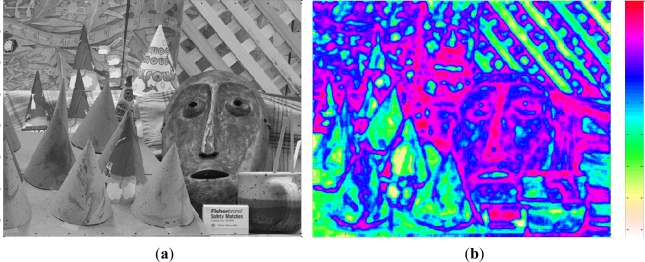
(**a**) Original image. (**b**) Corresponding entropy image.

Figure 4(a) shows a test image of 1,024 × 1,024 pixels with a captured scene presenting many different features and depths. Figure 4(b) depicts the processed entropy image. The greater the entropy of the image area represented, the highest color scale is shown at the right.

**Figure 5.**
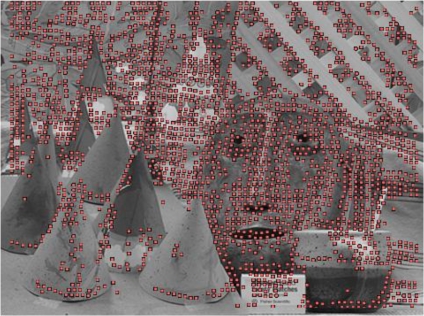
Entropy areas used as matching candidates.

Figure 5 shows the image pixels with higher entropy which are suitable to be used in the computation of stereo disparity considering a dispersion of 5 pixels between rows. It can be seen that suitable points are distributed throughout the whole image, generating a dense stereo image. Possible areas with few points are due to low entropy values. It is worth to note that in these areas ambiguous matches are obtained. Properly selecting different thresholds more or less pixels in the entropy image are selected. The adjustment of the threshold could be specified locally, considering different areas in the image each one with its own dynamic entropy threshold.

### 4.1. Hardware Block for Entropy Computation

**Figure 6.**
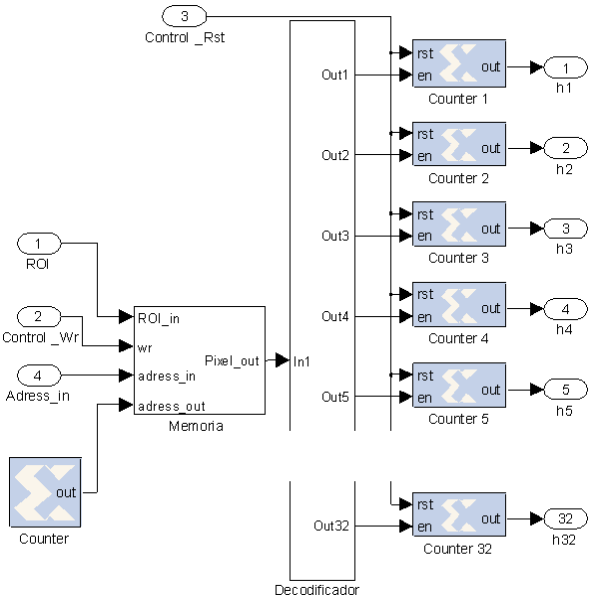
Histogram computation block.

As indicated in the previous section, the calculation of the entropy of a ROI image is obtained from its histogram (occurrences of each gray level). The proposed design process the image in real-time without the need to store the image in memory. Therefore, linear buffers are used to present the image stream in a parallelized way thus it is possible to access to all the pixels in the area at the same time. The aim is to process a new image area at each clock cycle. Considering a delay for each histogram computation of K cycles, it is necessary to use at least K histogram blocks to reuse the block once it has finished its computation. The scheme of one histogram calculation block (implemented in XSG) is shown in Figure 6.

**Figure 7.**
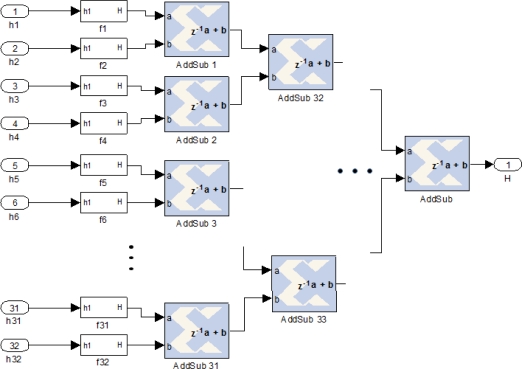
Entropy calculation from counter histogram ROI values.

Let us consider a squared ROI of size *n* by *n* pixels in the image. By using a dual-port memory it is possible to write all data from the input port in one clock cycle and then read each position, pixel by pixel, updating a specific counter block which is associated to the gray level of that pixel. The histogram division consider herein is 32 bins for the 256 gray levels, which provides enough information while reducing the hardware resources consumed. In addition, the number of bins has been reduced from the total number of gray levels in order to reduce the entropy measurement which in turn reduces the number of points with high entropy. The later selected points, considering this smoothed entropy, have more probability to get a correct value of disparity. The histogram values are introduced to a combinational block *f_i_* that implements Equation (4) in order to obtain the entropy values:

(4) $$f(x)=-H(x)\;{\text{log}}_{2}\;H(x)$$

where *H(x)* represents the histogram values related to the probability of occurrence in a given area. A LUT function *f*(*x*) is made in the implementation block to carry out the calculation of the logarithm as given in [22], achieving a maximum processing frequency up to 122 MHz. All resulting values of the *f_i_* functions are summed by a vertical line of adders in cascade as it is shown in Figure 7.

With the proposed structure, the entropy value for a given image ROI is obtained each clock cycle, after an initial latency. Thus, the hardware block provides a whole image entropy on-the-fly with minimum implementation in hardware [23]. Next section, consider that there are stored in local memory the information about ROI candidates selected from camera 1 with a high threshold value, introducing the delayed row from image data flow of camera 2. An important aspect to note is that ROI data in both memories are stored following the row order, very useful to restrict possible region of matching in the correlation block as it will be explained later.

## 5. Data Arbitration for an Efficient Data Flow to Correlation Block

Candidate points from image flow of camera 1 are stored in the double buffer alternating the memory for each new processed row. ROI values and other features as mean gray level, standard deviation, *etc.* could be introduced as the vector to be matched with camera 2. Data flow from camera 2 is delayed a row. Therefore, at the start of a row from camera 2, all the possible candidates from camera 1 are already stored in memory. Thus, each possible ROI selected from camera 2 data flow is offered to the correlator block in order to obtain a valid matching with any of the values stored in current memory used for camera 1 candidates.

**Figure 8.**
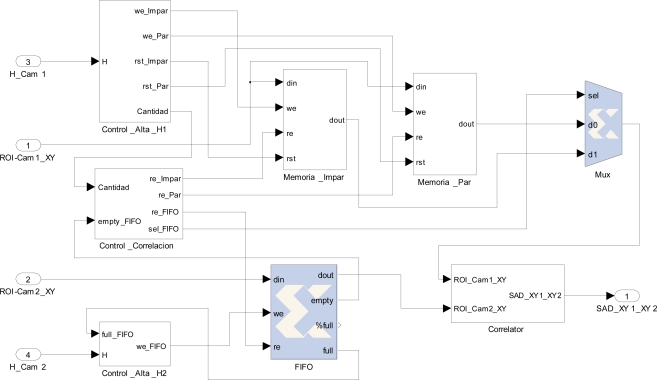
Control data flow to introduce data to correlator block.

Figure 8 shows an implementation diagram for data arbitration. Control_Alta_H1 subsystem determines whether the area under analysis exceeds the threshold level, if so the ROI values and location are written in local memory (Memoria_impar/par). The same procedure is done for image flow of camera 2 (Control_Alta_H2). In this case, the double buffer technique is substituted with a FIFO memory. If exists data in this FIFO memory, the correlator block starts the search around. It reads consecutively the candidates of camera 1 looking for a match with the current point of camera 2 obtained from the FIFO memory. Entropy value of the current ROI (H_Cam1) is introduced to the Control_Alta_H1 block, and compared with the corresponding threshold. If the value of entropy passes the threshold, the ROI is given in parallel to a FIFO memory. In the same way, the Control_Alta_H2 block comes with the value H_Cam2 and another FIFO memory for that image flow. Each ROI stored in those FIFOs is given to the correlation block. Next section presents the efficient correlation the range of possible ROIs for a given image point.

## 6. Efficient Stereovision Correlation

**Figure 9.**
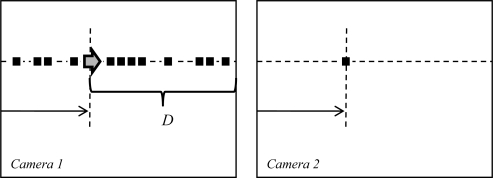
Range D available for correlation.

Several techniques could be applied to obtain the correlation matching. In our work, Sum of Absolute Difference (SAD) is used. The literature discusses several variations that use this method of correlation. Most focus on the selection of the ROI size and its geometric shape [24]. Geometry variation for the ROI image is out of the scope of this paper. SAD is suitable to be implemented in reconfigurable hardware, using simple efficiently available resources. In contrast, SSD (sum of squared differences) needs to include a multiplier block to calculate the correlation value. In our context, it is advisable to implement hardware blocks for an algorithm of reduced complexity which provides correct results. The SAD correlation for two image areas considering rectangular ROIs is obtained by the application of Equation (5):

(5) $$z(i,j)=\text{arg}\;{\text{min}}_{k\in D}\sum_{r=0}^{r=n-1}\sum_{q=0}^{q=m-1}\left|I_{L}\;\left(i-r,j-q+k\right)-I_{R}\left(i-r,j-q\right)\right|$$

where I_L_ and I_R_ are the set of pixels in the left and right image respectively; D is the number of consecutive pixels in the possible overlapping range where to find the correspondence of the I_R_ image in the I_L_ image. Therefore, each ROI stored in the FIFO is correlated only with the areas of selected buffer that are within the range D, processing the minimum number of possible correlations. This range is defined for each new area of the FIFO to correlate, taking into account the location of current camera 2 pixel. In Figure 9 it is shown an example of range D available for correlation given a specific pixel from camera 2.

**Figure 10.**
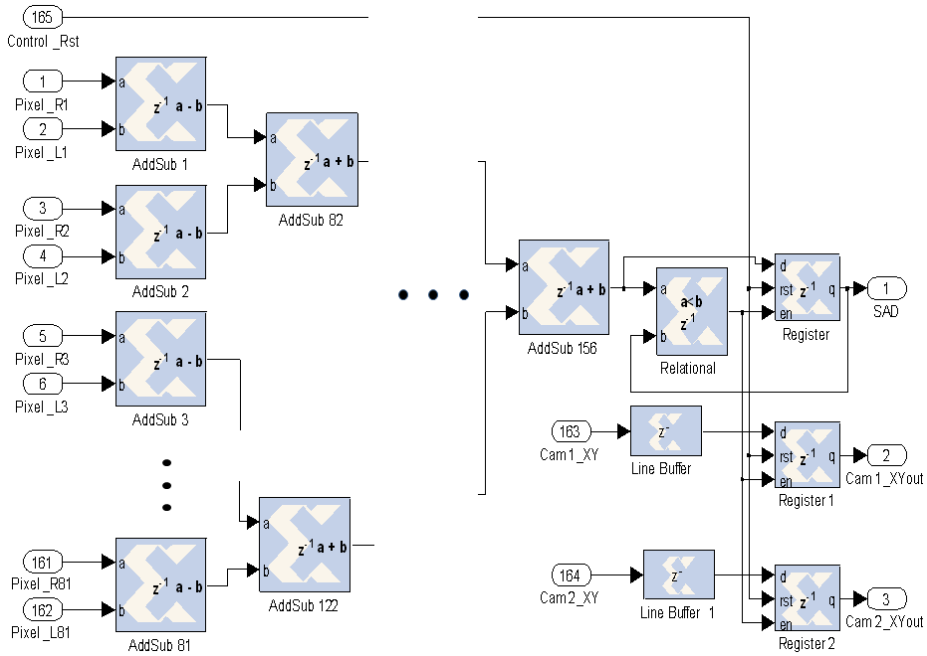
Correlation implementation.

To carry out the implementation of the correlation block, considering ROIs of *n* × *n* pixels to correlate, *n* × *n* subtract operations are needed. Next, considering the absolute value of each subtraction, a pipeline of adders implemented in cascade calculates the overall total sum. After an initial latency, a complete pair ROI correlation is made in each clock cycle. In Figure 10, the implementation for a ROI of 9 × 9 pixels is shown. Several stages of adders compute in pipeline the sum of absolute differences (SAD output). Besides the block offers synchronized the location of pixels in camera 1 and 2 to obtain the disparity.

As indicated above, all selected points from camera 1 should be good candidates in order to obtain a satisfactory match. But, possible ambiguities may occur if certain selected areas are too similar while searching in the same row (*i.e.*, textured zones) [23]. To solve these uncertainties, a hardware block to discriminate ambiguous points is proposed. On the other hand, possible errors due to occlusions will be correctly discarded because the matching provides low values of correlation thus discarding that ROI.

**Figure 11.**
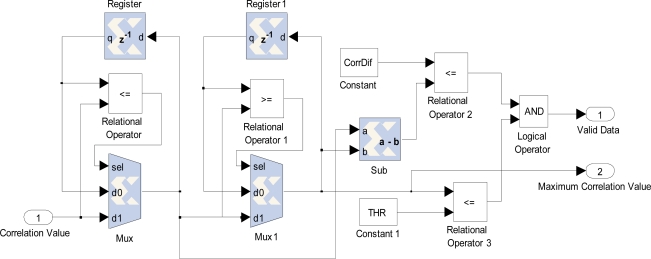
Implementation of ambiguous points discriminator.

For each ROI candidate from camera 2 a set of correlation values is obtained. The minimum SAD correlation value represents the highest similarity between areas, and therefore provides the suitable correspondence. There are two situations where the correspondence should be discarded. First, if the minimum SDA correlation value does not satisfy a minimum similarity threshold, and second, when several correlation values are very close to that minimum SDA correlation value, because several ROIs could be considered as a suitable matching (ambiguous points). For the latter, a hardware block is proposed (Figure 11). The two better correlation values are stored for each correlation sub-process. ROI candidates are discarded if there is not enough correlation difference (*CorrDif*) between them. The disambiguation block allows for pipelined processing.

## 7. Results

This section presents different results of the proposed SoC. First, a detailed analysis of all parameters involved in the algorithm is performed, obtaining the resource consumption for different configurations. Next subsection presents the synthesis results for a SoC implementation in a XC6SLX150T Xilinx Spartan 6 FPGA.

### 7.1. Analysis of Design Parameters

The fundamental requirement in the proposed SoC is real time image processing. To achieve this approach, the number of points to process should be adjusted properly, in order to not exceed the planned consumption of hardware resources. In the proposed algorithm, entropy thresholds control the number of candidate points to process and limit the maximum number of 3D points. Several options can be used to perform a dynamic control of these thresholds, as Gaussian estimators, PID regulation, *etc.* from an in-FPGA processor. Another design requirement is to obtain stereo points throughout the image, so a certain number of candidate points in each line should be established. In addition, taking into account the image size in order to megapixels (*Mpix*) provided by current sensors will be necessary to introduce scatter parameters in columns and rows.

**Figure 12.**
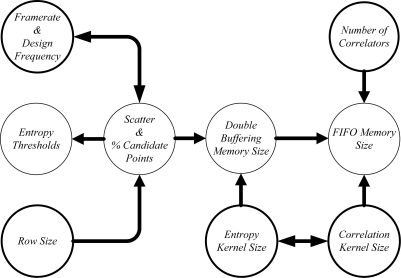
Relationship between parameters: Influence and dependence.

Figure 12 presents the relationship between all parameters involved in the proposed system. Highlighted parameters are independent; while the rest of parameters are fixed by means of an expression.

As indicated in previous sections, stereo 3D processing is carried out row by row, with a double buffering schema. Therefore, resources should be dimensioned to process a single row completely during the reception of the next image row. Moreover, assuming a continuous data transmission from the sensor, the design frequency should be fixed to at least the data transmission frequency (*camera framerate*).

To begin a correlation stage of a single row, all candidate points of camera 1 should previously be stored in a buffer memory. Therefore, only two parameters, framerate and row size, are involved to determinate the suitable number of candidate points to be processed in a row, through a dynamic threshold parameter. In some cases, different areas in the image could present different values of entropy so that the number of candidate points could vary considerably. To address this situation, the threshold 1 is an array of parameter values, presenting different value for each camera line. At the end of a frame processing, new values for threshold 1 are modified in order to obtain the desired number of candidate points. The constant *p* represents the ratio between the number of candidate points in the camera 2 and camera 1. In the proposed algorithm, the threshold parameter of the camera 2 is used to maintain stable this relationship, updated in each line in the same way that the threshold 1. A simple PID control loop should be capable to adjust the threshold values, but the parameters should be adjusted to have an overdamped oscillation. Besides, to obtain a distribution of points throughout a row, a scatter parameter is applied to obtain a minimum separation between candidate points.

**Figure 13.**
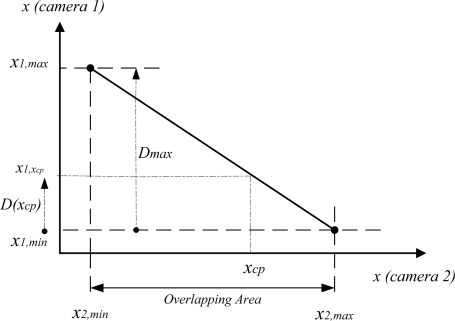
Range *D* in overlapping area.

Once the above parameters are fixed we could estimate the number of correlations to execute in a single row by means of expression Equation (6):

(6) $$n_{c/r}=\sum_{i=0}^{N}\left(\frac{{\mathrm{Size}}_{\mathrm{Row}}-D(x_{2})}{\mathrm{Sct}}\right),\;N=\left(p\;\frac{{\mathrm{Size}}_{\mathrm{Row}}}{\mathrm{Sct}}\right)$$

where *Size_Row_* is the number of pixels in an image row, *Sct* is the scatter applied to the row, *D* (*x*_2_) is the range of possible values of the camera 1 to process respect to a candidate point from camera 2, as shown in Figure 13. For example, given a point *X_CP_* in the row of the camera 2, the range of possible values in the row of the camera 1 is represented by this expression: *D*(*X_CP_*)={*x*_1,*min*_, *x*_1,*x*_*CP*__}.

The distance, between *x*_2,*min*_ and *x*_2,*max*_, represents the overlapping area where correspondences could locate. Therefore, this distance defines the maximum range expressed as *D_max_* in the Figure 13.

**Figure 14.**
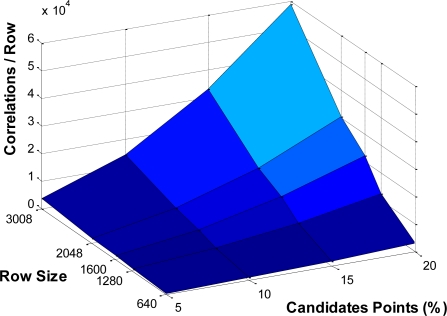
Example of estimated number of correlations for each row.

To clarify this requirements, in Figure 14 an estimated number of correlations to process in each row is shown. The y-axis represents the size of the row in pixels and the x-axis the percentage number of candidate points.

Two parameters define the size of the double buffer memory: entropy kernel size and number of candidate points to obtain 3D depth. It could store coordinates and area to process of all possible suitable points in a buffer, while in other buffer candidate points are correlated. This size *d_DBM_* could be estimated by Equation (7):

(7) $$d_{\mathrm{DBM}}=2N_{\mathrm{CP}}\;n^{2}$$

where *N_CP_* is the maximum number of suitable points that the entropy block could select through the current threshold and *n* is the size of the kernel in pixels, assuming a squared kernel. The number of suitable points is fixed through previous parameters. On the other hand, kernel size parameter is practically independent of processing time, completed initial latency whenever a new image is transmitted. In contrast, resources are increased in entropy and correlation blocks. Sometimes, according to image information, a greater kernel size improves the location of the correspondence and avoids some ambiguities. However, the FIFO memory must be larger since more points are suitable due to a lower threshold. The FIFO memory size is fixed through size of the double buffering memory and the parameter *p*, because this parameter relates the number of candidates points to store. Entropy and correlation kernels have the same dimension (*n* × *n*). In the proposed implementation, it will be possible to configure the correlation kernel less than entropy kernel in order to reduce resources.

Finally, the number of correlators to implement is analyzed. In previous sections, a single correlator has always been used in our SoC implementation. Depending on the resources available or framerate to process, several correlators could be implemented. The two main blocks (entropy block and correlation block) process an area at each clock cycle, after the initial latency of each block. As it is shown in Figure 14, the number of correlations exceeds the maximum number of pixels to process in the entropy block (all pixels in the row). So the next processing row should wait for the correlation block to finish its task. Otherwise, it just could discard the remaining candidate points in the correlation process, losing some 3D points. To increase the achievable framerate, the optimal solution would be to implement several correlator blocks in order to ensure complete processing of points in the ROI matching.

Table 1 shows the values of all parameters for different configurations. The first column contained common image sizes used in the actual systems. We make a distinction between various values of candidate points and two ROI sizes. From this, all parameters are determined.

### 7.2. SoC Implementation Results

In Tables 2 and 3 the resources consumed by the SoC implementation using a XC6SLX150T Xilinx Spartan 6 FPGA are shown.

**Figure 15.**
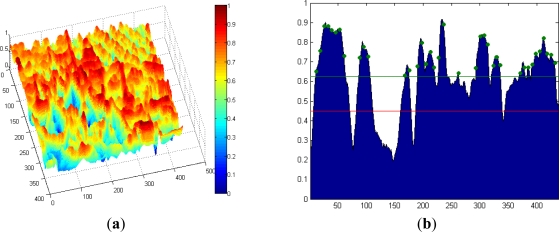
(**a**) Entropy values for the Cones dataset. (**b**) Entropy values for horizontal line 200.

Figure 15(a) shows the results of entropy for the Cones dataset. To obtain these results, the size of the ROI is set to 13, using 32 bins. A particular case is shown in Figure 15(b), which represent, the values of entropy for a specific line of the image (line 200). The thresholds for determining whether a suitable point is selected are displayed with green color. These thresholds are always above the minimum threshold shown in red. The green circles represent selected points to be stored in memory. The percentage of candidate points to be selected is set to 15% and the dispersion value to 7. It is observed that in areas of the line where entropy values are lower than the threshold are not selected points, so the number of selected points is below 15%. In successive iterations, the threshold should decrease in these areas in order to obtain more points (≈15%).

**Figure 16.**
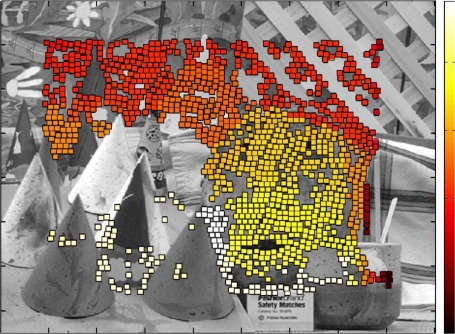
Dense disparity map.

After presenting different possibilities of the system, a specific configuration has been tested, considering an image size of 1,280 × 960 pixels. The ROI size was set to 9 × 9 pixels, same kernel size for the calculation of entropy and correlation matching. Desired candidate points was set to 10% of points for each row, and 4 pixels dispersion. Figure 16 shows the dense disparity map with 3D information recovered from the image tests.

The stereo flow is injected directly from an external memory connected to the FPGA. This real emulation allows considering any stereo system regardless of the source image used, without taken care about other aspects such as control of camera lines and parameters of image capture. This dense map could give up to 30,000 data points using the configuration presented above.

**Figure 17.**
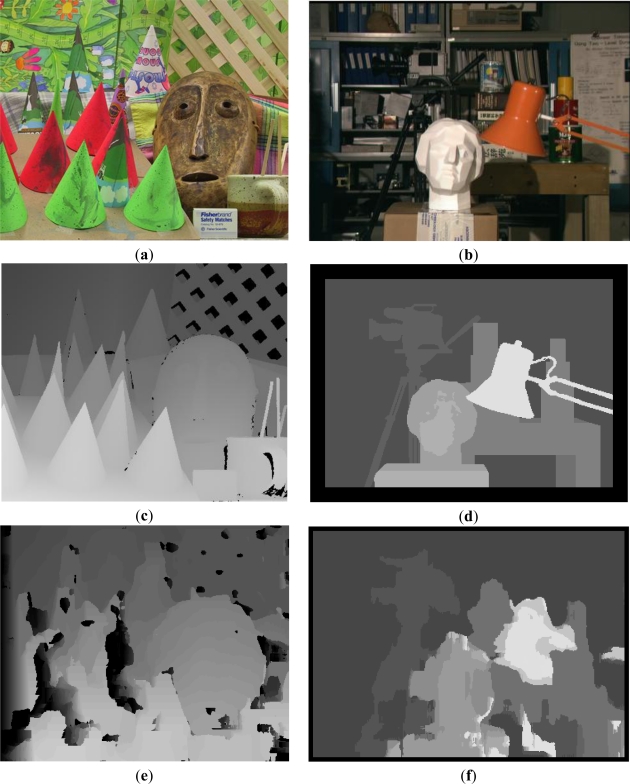
Correlation results: (**a**) Original Cones dataset. (**b**) Original Tsukuba dataset. (**c**) and (**d**) Ground truth. (**e**) and (**f**) Correlation Results.

To finalize the results section, Figure 17(e,f) shows overall correlation results for the Cones and Tsukuba datasets without taking into account the selected suitable points, respectively. So these results are provided by the proposed SoC in the case that all points of the image are suitable points. This leads have zero entropy threshold. In Figure 17(a,b) the original images of each dataset are shown. Then, it shows the ground truth (Figure 17(c,d)). Depending on the parameters configuration, a number of disparity points are provided by the SoC. They should have an entropy value exceeds its threshold. Thus, appropriate disparity values will be obtained while maintaining accurate results.

## 8. Conclusions

This paper presents an efficient hardware implementation of a parametrisable stereovision SoC. The main contribution is the proposal of hardware architecture to correctly recover stereovision 3D measurements distributed throughout the whole image, eliminating the limitation on the maximum number of stereo correspondence points as it is commonly considered in many applications. Using double buffering techniques properly combined with pipelined processing, the use of reconfigurable hardware achieves a parametrisable SoC which gives the designer the opportunity to decide the correct dimension and features of this coprocessor.

Control parameters could be downloaded into the FPGA via any communication bus (PCI, Ethernet) or changed by the later stages of the algorithm by means of an embedded processor. Parameters suitable for change are threshold values for candidate selection and dispersion of ROI candidates. Current digital cameras offer the possibility of modifying the frame rate reducing the image processed, thus high level computer vision may modify the number of 3D data to obtain for each particular configuration of cameras in the stereovision system. Other SoC parameters are fixed and a reconfiguration of the hardware should be addressed in those cases, *i.e.*, image size, histogram bins, gray levels, *etc.* The SoC may return the number of total candidate points in each row or complete image flow, the number of ambiguous candidates in each row or complete image, *etc.* It is possible to process stereo images in real-time, thus, considering a hardware clock of 100 MHz inside the FPGA, image flows up to 50 fps of dense stereo maps (10% of pixels) could be obtained for 2 Mpix images, with a minimum initial latency. Compared with time-of-flight depth cameras, classical stereovision may be used with any standard camera which in turn reduces the cost of the embedded system.

The implementation of computer vision algorithms on reconfigurable hardware, explicitly low level processing, opens up the prospect of its use in autonomous systems, and they can act as a coprocessor to reconstruct 3D images with high density information.

## Figures and Tables

**Table 1.** Design parameters and resource consumption.

**Size Image (Mpix)**	**Row Size (Pix)**	**Column Size (Pix)**	**Candidate Points**	**ROI Size (*n* × *n*)**	**Correlations/Row**	**Double Buffer Memories Size (KBytes)**	FIFO Memory Size * (KBytes)	**Delay Buffers (KClycles)**	Framerate * (Images/Sec) fclk = 100 MHz
0.3	640	480	5%	9 × 9	203	5	3	5	4,203
				13 × 13		10	7	8	4,203
			10%	9 × 9	781	10	7	5	1,090
				13 × 13		21	15	8	1,090
			15%	9 × 9	1,781	15	11	5	476
				13 × 13		32	24	8	476
1.2	1,280	960	5%	9 × 9	781	10	7	11	545
				13 × 13		21	15	16	545
			10%	9 × 9	3,125	20	15	11	135
				13 × 13		43	32	16	135
			15%	9 × 9	7,031	31	23	11	60
				13 × 13		64	48	16	60
2	1,600	1,200	5%	9 × 9	1,416	12	9	14	225
				13 × 13		27	20	20	225
			10%	9 × 9	5,583	25	18	14	57
				13 × 13		54	40	20	57
			15%	9 × 9	12,500	38	28	14	25
				13 × 13		81	60	20	25
3	2,048	1,536	5%	9 × 9	1,904	16	12	18	143
				13 × 13		34	25	26	143
			10%	9 × 9	7,617	33	24	18	35
				13 × 13		69	51	26	35
			15%	9 × 9	17,139	49	36	18	15
				13 × 13		103	77	26	15
5.3	3,008	1,960	5%	9 × 9	3,650	24	18	27	62
				13 × 13		50	37	39	62
			10%	9 × 9	14,602	48	36	27	15
				13 × 13		101	75	39	15
			15%	9 × 9	58,679	73	54	27	3
				13 × 13		152	114	39	3

**Table 2.** Resources used on a XC6SLX150T: Slice Registers and Slice LUTs.

	***XC6SLX150T Xilinx Spartan 6 FPGA***			
**ROI Size (*n* × *n*)**	**# Resources Entropy Block**		**# Resources Correlation Block**	
	**SliceRegisters**	**SliceLUTs**	**SliceRegisters**	**SliceLUTs**
9 × 9	21%	46%	5%	11%
13 × 13	30%	65.9%	7.5%	16.4%

**Table 3.** Resources used on a XC6SLX150T: Block RAM.

**Image Size (Mpix)**	**Row Size (Pix)**	**Column Size (Pix)**	**Candidate Points**	**ROI Size (*n* × *n*)**	**# Utilization Block *XC6SLX150T-*RAM/FIFO**
0.3	640	480	5%	9 × 9	1 %
				13 × 13	2 %
			10%	9 × 9	2 %
				13 × 13	5 %
			15%	9 × 9	4 %
				13 × 13	9 %
1.2	1,280	960	5%	9 × 9	2 %
				13 × 13	5 %
			10%	9 × 9	5 %
				13 × 13	12 %
			15%	9 × 9	8 %
				13 × 13	18 %
2	1,600	1,200	5%	9 × 9	3 %
				13 × 13	7 %
			10%	9 × 9	7 %
				13 × 13	15 %
			15%	9 × 9	10 %
				13 × 13	23 %
3	2,048	1,536	5%	9 × 9	4 %
				13 × 13	9 %
			10%	9 × 9	9 %
				13 × 13	19 %
			15%	9 × 9	14 %
				13 × 13	29 %
5.3	3,008	1,960	5%	9 × 9	6 %
				13 × 13	14 %
			10%	9 × 9	13 %
				13 × 13	29 %
			15%	9 × 9	21 %
				13 × 13	44 %
